# Applicability of metabolomics in axial spondyloarthritis: a scoping review

**DOI:** 10.1186/s42358-026-00562-y

**Published:** 2026-08-06

**Authors:** Denise Maiara Silva Carneiro, Déborah Catharina Campos Siqueira, Ricardo Oliveira da Silva, Claudia Diniz Lopes Marques

**Affiliations:** 1 Programa de Pós-graduação em Biologia Aplicada à Saúde (PPGBAS) – Universidade Federal de Pernambuco, Recife, PE Brasil; 2 Programa de Pós-graduação em Química (PPGQ) – Universidade Federal de Pernambuco, Recife, PE Brasil; 3 Hospital das Clínicas/Empresa Brasileira de Serviços Hospitalares, Universidade Federal de Pernambuco, Recife, PE Brasil

**Keywords:** Biomarkers, Early diagnosis, Therapeutic response, Pathophysiology

## Abstract

**Objective:**

This scoping review aims to explore the applicability of metabolomics in identifying biomarkers for early diagnosis and disease monitoring in patients with axial spondyloarthritis (axSpA).

**Methods:**

We conducted a scoping review following the PRISMA-ScR and JBI guidelines. Searches were carried out on the Web of Science, Scopus, and MEDLINE databases. After applying the eligibility criteria, 13 articles were included in the final analysis.

**Results:**

Specific metabolic signatures, mainly amino acid, fatty acid, and energy and lipid metabolism metabolites, were identified as potential tools for distinguishing axSpA patients from healthy controls and assessing treatment response.

**Conclusion:**

Metabolomics holds considerable promise for advancing the understanding of axSpA pathophysiology and facilitating biomarker discovery. However, further validation in diverse populations and methodological standardization are required before these findings can be translated into clinical practice.

## Introduction

Axial spondyloarthritis (axSpA) is a chronic inflammatory disease that affects the axial skeleton and sacroiliac joints; it is characterized by the insidious onset of low back pain and is often associated with progressive structural and functional impairments, leading to decreased quality of life [1]. The global prevalence of axSpA is approximately 1%, with considerable geographic variability, largely attributed to the distribution of the human leukocyte antigen B27 (HLA-B27) [2]. Documented evidence indicates that axSpA is more common in men (3:1), with a typical onset in the third decade of life and almost exclusively before the age of 45 [3].

In recent years, significant progress has been made in understanding the pathogenesis of axSpA, including associations with HLA-B27 [4] and both MHC and non-MHC genes (major histocompatibility complex) [5]. Despite these advances, many mechanisms remain unclear, highlighting the need for more comprehensive investigative approaches. In this context, the identification of new biomarkers through advanced, high-precision techniques, particularly metabolomics, is crucial for enhancing the understanding of the disease and informing therapeutic strategies.

Metabolomics refers to the comprehensive analysis of low-molecular-weight metabolites within specific biological systems [6]. Metabolites, in turn, represent the outcome of dynamic interactions among genes, proteins, and the environment [7–9]. Methods such as nuclear magnetic resonance (NMR) spectroscopy and mass spectrometry (MS) are often used in combination with gas or liquid chromatography for detailed metabolic analysis in clinical studies [10–12].

Recent studies have demonstrated the utility of metabolomics in identifying specific biomarkers for axSpA. For example [13], used NMR to identify serum metabolic profiles in axSpA patients, revealing metabolic signatures useful for early diagnosis and disease monitoring. Additionally, metabolomics has shown efficacy in distinguishing between rheumatic diseases by identifying disease-specific metabolites, particularly those involved in amino acid and lipid metabolism [12]. Some studies have also explored the link between the gut microbiota and metabolic alterations, suggesting potential diagnostic signatures that differentiate patients from healthy controls.

Given the impact of axSpA on patient function and quality of life, early diagnosis and accurate assessment of disease severity are essential for effective therapeutic interventions. This review aims to explore the applicability of metabolomics as a tool for identifying biomarkers for the early detection and monitoring of axSpA, synthesizing findings on metabolic profile differences between patients and controls, as well as dynamic metabolite changes before and after treatment.

## Methods

This study is a scoping review structured according to the Joanna Briggs Institute methodology for scoping reviews [14] and the International Guidelines of the Preferred Reporting Items for Systematic reviews and Meta-Analyses extension for Scoping Reviews (PRISMA-ScR) [15]. Scoping reviews are increasingly used in health sciences to assess emerging evidence, clarify concepts or definitions, and analyze how research is conducted in each field [16]. As it is based on publicly available data, this study did not require ethics committee approval.

The review was conducted between October and November 2024 and followed five main steps: (1) formulating the research question; (2) defining inclusion and exclusion criteria based on relevance to the topic; (3) developing tailored search strategies for each database; (4) selecting and extracting data from the included studies; and (5) critically analyzing the evidence and presenting results.

The research question was formulated via the PCC framework: P (population): patients with axial spondyloarthritis; C (Concept): application of metabolomics to analyze metabolic profiles; C (context): use of metabolomics in biological samples (serum, plasma, urine, feces, or tissue) in the context of axSpA. The final research question was as follows: What is the potential of metabolomics to identify novel biomarkers for the early diagnosis and monitoring of axial spondyloarthritis?

The inclusion criteria were as follows: (a) studies focused on metabolomics in the context of axSpA; (b) full texts were available; and (c) original research articles were included. The exclusion criteria were as follows: (i) studies not primarily addressing metabolomics in axSpA patients or lacking relevant data; and (ii) publication types such as reviews, conference abstracts, news, editorials, retractions, letters, or commentaries.

Searches were conducted in MEDLINE via PubMed, the Web of Science Core Collection, and SCOPUS. The search strategy included terms such as “Metabolomics”, “Metabolic Profiling”, “Metabolome”, “Biomarkers”, and “Axial Spondyloarthritis” or “Ankylosing Spondylitis”, with Boolean operators adapted to each database.

Two reviewers independently screened studies based on the inclusion and exclusion criteria. After the initial search, duplicate records were removed via Zotero, and selection was managed via Rayyan (Qatar Computing Research Institute). Initially, studies were screened by title and abstract, followed by full-text review for final inclusion.

For data extraction, a synoptic chart was utilized based on the JBI manual, which included the following elements: author, journal, year, country, study objective, methodology, population, metabolomic technique, main findings, conclusions, and theoretical frameworks. Quality assessment was not conducted, as it is not customary in scoping reviews.

The results are presented in summary, while the discussion integrates the key findings, acknowledges limitations, and explores clinical implications.

## Results

### General characteristics

A total of 201 publications were initially identified. After 47 duplicates were removed, 154 articles remained for title and abstract screening. Of these, 26 articles were assessed in full, and 13 were included in this scoping review. The characteristics of the included studies are summarized in Table 1. Notably, the term axSpA here refers to various clinical forms, including ankylosing spondylitis (AS), reactive arthritis (ReA), and undifferentiated spondyloarthritis (uSpA).

Studies were published between 2016 and 2024, with a concentration in 2019 and 2021, and were conducted primarily in Asia, particularly in China (*n* = 9) and India (*n* = 4). Observational designs predominated, especially cross-sectional and case–control studies, whereas two investigations were classified as clinical trials. The total sample comprised 1,400 participants.

Considerable heterogeneity was observed regarding participant characteristics. Most cohorts were predominantly male (7, 8, 11), reflecting the epidemiological profile of axSpA and related conditions. Disease activity was assessed using different clinical and laboratory measures, including BASDAI, ASDAS, CRP, and ESR. Disease duration varied substantially across the included studies, ranging from 6 months to 14.2 ± 4.8 years [7, 16]. In addition, some investigations stratified participants according to disease duration or disease stage [11, 12].

Nine studies compare the metabolic profiles of axSpA patients and healthy controls. In the remaining studies, additional disease control groups were included, comprising patients with rheumatoid arthritis (RA) and/or osteoarthritis (OA), which served as inflammatory and non-inflammatory controls, respectively [10, 11, 17, 18]. Only two studies investigated metabolomic changes associated with therapeutic interventions [8, 13]. In the remaining studies, potential treatment-related effects on metabolomic profiles were minimized by implementing medication washout periods prior to sample collection [10, 16, 19, 20] or by recruiting treatment-naïve patients [7, 8, 11, 21].

The biological samples analyzed included serum, synovial fluid [17–19], feces [10, 20], and plasma, urine, and ligament tissue [7]. The techniques used include ¹H-nuclear magnetic resonance (NMR) [7, 10, 13, 17, 19], liquid chromatography‒mass spectrometry (LC‒MS) [8, 11, 21, 23, 24] and gas chromatography‒mass spectrometry (GC‒MS) [12, 18, 20].

**Table 1:** Articles included in the scoping review

Authors/Year	Country	axSpA clinical characteristics	Control Group	Sample Type	Analytical Technique	Main Findings
Wang et al. (2016)	China	74 axSpA (AS) Plasma/Urine 44 (38 M, 6 F) BASDAI: 3.2 30 (26/M/4F) BASDAI: 5.8	44 HC Plasma/Urine 44 (38 M, 6 F) 30 (19 M/11F)ligament tissue	Plasma, urine, ligament tissue surrounding the hip joint.	NMR	Twenty potential metabolites are indicative of AS, including leucine, valine, glycerol, acetoacetate, 3-hydroxybutyrate, triglycerides, creatinine, and choline. Alterations related to lipid, glucose, and choline metabolism were observed in AS patients compared to controls, showing good discriminatory capacity between AS and controls.
Shao et al. (2016) [10]	China	40 axSpA (24 M/16F) Inactive disease	34 HC (19 M/15F) 35 RA (10 M/25F) Inactive disease	Feces	NMR	Decreased levels of butyrate, propionate, methionine, and hypoxanthine; increased taurine, methanol, fumarate, and tryptophan in axSpA/RA vs. controls. No significant differences between axSpA and RA.
Ahmed et al. (2019)	India	32 axSpA (25 M/7F) ≥ 1 swollen joint with effusion	18 HC (17 M/1F)	Serum; synovial fluid	NMR	Distinct metabolic profile in serum and synovial fluid of ReA/uSpA compared to HC; differences in amino acids, fatty acids, and carbohydrate metabolites; increased Phe/Tyr in ReA and axSpA vs. RA/OA; similar profiles between ReA and axSpA.
He et al. (2019)	China	49 axSpA (26 M/23F) BASDAI: 3.7	38 HC (20 M/18F)	Feces	GC-MS	Fecal metabolites differ between SpA and HC; 19 differential metabolites identified, with sex-specific variations linked to clinical differences.
Muhammed et al. (2019) [17]	India	30 axSpA (ReA, uSpA) (24 M/6F)	25 RA; 21 OA sex not reported	Serum; synovial fluid	NMR	Increased Phe/Tyr ratio in synovial fluid in ReA/axSpA vs. controls and RA/OA; higher Phe in ReA/axSpA.
Zhou et al. (2020) [11]	China	30 axSpA (20 M/10F) ASDAS-CRP: >2.1	30 HC (16 M/14F) 32 RA (14 M/18F)	Serum	LC-MS	4-Hydroxy-L-proline, alanine, γ-aminobutyric acid (GABA), glutamine, and taurine were identified as candidate biomarkers in SpA and RA vs. controls. Alanine, aspartate, and glutamate metabolism were elevated; lysine, proline, and serine correlated with disease activity in axSpA.
Dubey et al. (2021) [18]	India	58 axSpA (ReA) (49 M/9F) Acute knee arthritis with effusion	21 RA (4 M/17F) 20 OA (4 M/16F)	Synovial fluid	NMR	Elevated levels of alanine, lactate, and choline in the synovial fluid of ReA. Active inflammation; distinct profiles of RA and OA.
Ou et al. (2021)	China	32 axSpA (29 M/3F) ASDAS-CRP: 4.36 Anti-TNF therapy	40 HC (37 M/3F)	Serum	LC-MS	Fifty-five differential metabolites were identified; a five-metabolite panel showed high diagnostic accuracy. TNF inhibitors modulated 21 metabolites linked to pathogenesis and treatment. Several phosphatidylcholines and lysophosphatidylcholines decreased in the serum of patients with axSpA. Following treatment, LPCs decreased the pathological ceramides were decreased.
Gupta et al. (2021)	India	81 axSpA (71 M/10F) ASDAS-CRP: 2.3 17 patients reassessed at 3 months (NSAID treatment)	86 HC (72 M/14F)	Serum	NMR	Increased amino acids and N-acetyl glycoproteins; decreased LDL, VLDL, polyunsaturated lipids, and acetone in axSpA vs. controls. Therapy reduced inflammatory amino acids and N-acetyl glycoproteins (NAG).
Li et al. (2022) [23]	China	115 axSpA (71 M/44F) ASDAS: 2.05	108 H (33 M/75F)	Serum	LC	Elevated triacylglycerol (18:0/18:1/20:0) and decreased levels of phosphatidylcholine (16:0 e/26:4 and 18:1/22:6) in axSpA vs. controls.
Guo et al. (2023)	China	57 axSpA (28 M/29F) 17 acute (elevated) 40 remission	40 HC (20 M/20F)	Serum	GC-MS	The metabolic signature of AS was characterized by alterations in amino acid, energy, and lipid metabolism. Acute-stage patients exhibited lower levels of fatty acids, glucose, and amino acids than patients in remission. A biomarker model based on 2-hydroxybutanoate and hexadecanoate accurately distinguished between disease stages.
Li et al. (2023) [21]	China	20 axSpA (12 M/8F) ASDAS-CRP: 2.35	20 HC (10 M/10F)	Serum	LC -MS/MS	A nine-metabolite panel distinguished healthy individuals from AS patients, highlighting reduced dipeptides, indicative of altered protein metabolism, and elevated anti-angiogenic fibrinopeptides, suggesting functional relevance as a diagnostic biomarker.
Li et al. (2024) [24]	China	67 axSpA (44 M/23F) Inflammatory markers (ESR 23.68 mm/h; CRP 22.34 mg/L	67 HC (44 M/23F)	Serum	LC - MS/MS	Twenty-six metabolites exhibited high sensitivity and specificity for distinguishing patients with AS from healthy HCs. Among them, 3-amino-2-piperidone, hypoxanthine, and octadecylamine showed excellent discriminatory ability and were identified as promising biomarkers for AS.

AS, Ankylosing Spondylitis; ASDAS, Ankylosing Spondylitis Disease Activity Score; ASDAS-CRP, Ankylosing Spondylitis Disease Activity Score based on C-reactive Protein; axSpA, Axial Spondyloarthritis; BASDAI, Bath Ankylosing Spondylitis Disease Activity Index; ESR, Erythrocyte Sedimentation Rate; GABA, Gamma-Aminobutyric Acid; GC–MS, Gas Chromatography–Mass Spectrometry; HC, Healthy Controls; HLA-B27, Human Leukocyte Antigen B27; LC–MS, Liquid Chromatography–Mass Spectrometry; LDL, Low-Density Lipoprotein; LPCs, Lysophosphatidylcholines; M/F, Male/Female; MS, Mass Spectrometry; NMR, Nuclear Magnetic Resonance; NSAIDs, Nonsteroidal Anti-Inflammatory Drugs; OA, Osteoarthritis; PCs, Phosphatidylcholines; Phe/Tyr, Phenylalanine/Tyrosine; RA, Rheumatoid Arthritis; ReA, Reactive Arthritis; TNF, Tumor Necrosis Factor; uSpA, Undifferentiated Spondyloarthritis; VLDL, Very-Low-Density Lipoprotein.

### Metabolomic signatures of axSpA patients

axSpA is a complex disorder arising from the dysregulation of various metabolic pathways. Patients with axSpA present distinct metabolic profiles, particularly carbohydrate, lipid, and amino acid metabolism [22], which contribute to disease pathogenesis and activity.

An increase in citric acid cycle characterizes carbohydrate-related metabolic changes intermediates, such as fumarate and malonate, which inhibit glucose, glyceraldehyde, and succinate dehydrogenase [7, 10, 17]. In addition, increased lactate levels indicate alterations in anaerobic glucose metabolism [13]. Increased carbohydrate metabolites such as mannose and glucuronic acid have been detected in serum and fecal samples [17, 20]. Conversely, patients in remission have lower serum glucose levels than healthy controls [12].

Changes in lipid components, such as increased serum glycerol (a product of lipolysis) and divergent triglyceride levels between plasma and ligament tissue, suggest an imbalance in lipid utilization and storage [7, 19]. In addition, decreased serum levels of low-density lipoprotein (LDL) and very-low-density lipoprotein (VLDL) have been reported in axSpA patients [13, 20]. Fecal metabolomic analyses also identified reduced cholesterol levels in patients with axSpA.

Short-chain fatty acids (SCFAs), including acetate, propionate, and butyrate, showed altered concentrations across biological fluids, with increased serum acetate levels and decreased urinary and fecal levels [7, 10, 20]. Triacylglycerols and phosphatidylcholines were identified as potential biomarkers of axSpA [23]. Additionally, serum 2-hydroxybutanoate and hexadecanoate distinguished patients with active disease from those in remission [12].

Nine metabolites, characterized by reduced dipeptides and increased antiangiogenic fibrinopeptides, distinguished healthy individuals from patients with axSpA, suggesting potential diagnostic relevance [21]. Additionally, twenty-six metabolites differentiated AS patients from healthy controls, with 3-amino-2-piperidone, hypoxanthine, and octadecylamine highlighted as potential biomarkers [24].

Specific amino acids, such as lysine, proline, serine, and alanine, are correlated with disease activity [11]. Moreover, 55 differentially abundant metabolites were identified in patients with axSpA, with most amino acids exhibiting elevated serum levels in affected individuals [8]. Elevated levels of amino acids and N-acetyl glycoproteins are consistently found in axSpA patients [13]. These metabolites differentiated patients from controls and indicated potential diagnostic and monitoring utility.

Creatine and creatinine are imbalanced in axSpA patients, with increased serum creatine and decreased creatinine, suggesting that alterations in muscle and renal metabolism are influenced by systemic inflammation [11, 13]. Choline metabolism also differs by sample type and disease state [7, 10].

### Metabolomic profiling of spondyloarthritis subtypes and other arthritides

Several studies have compared axSpA with other inflammatory diseases to identify distinctive biomarkers. Zhou et al. [11] highlighted metabolites such as 4-hydroxy-L-proline, alanine, gamma-aminobutyric acid (GABA), glutamine, and taurine as potential biomarkers to distinguish axSpA patients and rheumatoid arthritis (RA) patients from controls. Additionally, elevated levels of alanine, lactate, and choline were detected in the synovial fluid of patients with reactive arthritis (ReA), indicating active inflammation and metabolic alterations distinct from those observed in RA [19].

Reduced levels of short-chain fatty acids (butyrate and propionate), methionine, and hypoxanthine, alongside increased taurine, methanol, fumarate, and tryptophan, were observed in patients with axSpA and RA, indicating that metabolic alterations are linked to inflammation and gut dysbiosis [10].

Synovial fluid analysis revealed a higher phenylalanine/tyrosine (Phe/Tyr) ratio in reactive arthritis patients and axSpA patients than in RA patients and osteoarthritis (OA) patients [18]. These findings suggest a specific amino acid metabolism signature in axSpA patients. However, similar profiles were identified in both the serum and synovial fluid of patients with reactive arthritis and those with axSpA, highlighting the variability in the results [17].

### Metabolomics reveals the drug’s mechanism of action

Therapeutic interventions, especially TNF (tumor necrosis factor) inhibitors, modulated metabolic profiles in axSpA patients. A normalization of 21 metabolites was observed posttreatment, indicating recovery of metabolic pathways. Amino acid regulation and glutaminolysis recovery are associated with clinical improvement [8].

Gupta et al. [13] reported posttreatment changes in glucose, LDL, polyunsaturated fatty acid, isoleucine, glutamic acid, and glycine levels, suggesting partial correction of metabolic abnormalities.

## Discussion

Advances in metabolomics have enhanced the understanding of axSpA pathophysiology, highlighting the potential of metabolic signatures as diagnostic, prognostic, and therapeutic tools. The metabolic profile of patients with axSpA is characterized by alterations in carbohydrate, lipid, and microbiota-related metabolism. Elevated levels of glycolytic intermediates and tricarboxylic acid (TCA) cycle metabolites suggest metabolic reprogramming of activated immune cells and increased energy demands during chronic inflammation. Lipid abnormalities, including alterations in glycerol, triglycerides, LDL, VLDL, and cholesterol levels, further support the involvement of metabolic pathways in both inflammation and cardiovascular risk. In addition, imbalances in short-chain fatty acids (SCFAs) underscore the immunomodulatory role of gut microbial metabolism.

The reviewed studies indicate that metabolic alterations in axSpA affect both immune-related and non-immune pathways. Alterations in methionine and N-acetylglycoproteins (NAG) have been associated with immune dysregulation and inflammatory activity, while disturbances in mannose, tryptophan, and butyrate metabolism further implicate immune regulation, host–microbiota interactions, and inflammatory responses in disease pathogenesis [7, 11, 14]. Conversely, reduced glucose utilization, increased ketone body production, triglyceride accumulation, and alterations in fatty acid β-oxidation and phospholipid metabolism point to broader disruptions in energy homeostasis and tissue metabolism [9, 13, 15, 16]. Consistent with these findings, Li et al. [22] reported increased triacylglycerol and decreased phosphatidylcholine levels, further supporting a role for lipid dysregulation in disease progression.

Collectively, the available evidence suggests that metabolic dysregulation in axSpA is not merely a consequence of chronic inflammation. Alterations in amino acid, lipid, carbohydrate, and microbiota-derived metabolites indicate an underlying immunometabolic imbalance that may contribute to disease pathogenesis. Their consistent association with disease activity and treatment response further highlights their potential utility as biomarkers for diagnosis, patient stratification, and therapeutic monitoring. Thus, metabolomics contributes to a deeper understanding of the biological mechanisms underlying axSpA while offering promising opportunities for the development of precision medicine approaches.

Zhou et al. [11] reported strong correlations between disease activity, as measured by ASDAS-CRP, and metabolites such as lysine, proline, alanine, and serine, indicating that metabolic alterations closely reflect clinical disease status. Likewise, Guo et al. [12] showed that a biomarker model based on 2-hydroxybutanoate and hexadecanoate accurately discriminated acute and remission stages of ankylosing spondylitis. Together, these findings highlight the potential of metabolomic biomarkers for assessing disease activity, monitoring disease progression, and supporting patient stratification.

In the therapeutic context, the normalization of 21 metabolites following TNF inhibitor treatment [8] highlights the close interplay between inflammatory pathways and metabolic homeostasis. These findings suggest that metabolic disturbances are dynamically linked to treatment response and may actively contribute to disease progression.

Moreover, metabolic similarities have been identified between axSpA and other autoimmune inflammatory diseases, particularly rheumatoid arthritis. In this context, Shao et al. [10] identified shared metabolic signatures between axSpA and RA in fecal samples. Similarly, Ahmed et al. [17] reported an increased Phe/Tyr ratio in the serum and synovial fluid of both ReA patients and uSpA patients, suggesting comparable metabolic profiles between these conditions.

Gender and subgroup differences also warrant attention. He et al. [20] reported that although fecal metabolic signatures were more pronounced in men, disease-related changes outweighed sex differences. These observations support the potential of noninvasive biomarkers for diagnosis and monitoring while highlighting the importance of investigating individual factors that influence metabolic profiles in patients with axSpA.

Although the included studies consistently identified alterations in amino acid, lipid, and energy metabolism, other potentially relevant metabolic pathways remain largely unexplored in axSpA. These include mitochondrial metabolism, one-carbon metabolism, nucleotide metabolism, and tricarboxylic acid (TCA) cycle-related pathways, all of which have been implicated in immune cell activation, oxidative stress, and chronic inflammatory diseases [6, 9, 22]. Although their specific role in axSpA remains poorly understood, these pathways represent important gaps in the current literature. Further investigation may provide a more comprehensive understanding of the metabolic mechanisms underlying axSpA and contribute to the identification of novel biomarkers and therapeutic targets.

The robustness and generalizability of the current evidence should be interpreted with caution. Although the included studies collectively enrolled approximately 1,400 participants, sample sizes varied considerably across investigations, with several studies involving relatively small cohorts. Importantly, all studies were conducted in Asian populations, predominantly in China and India, and largely comprised male participants, which may restrict the applicability of these findings to other ethnic and geographic populations. Additional sources of heterogeneity included differences in disease activity, disease duration, biological matrices, and metabolomic platforms. Such variability may partly account for differences in the metabolic profiles reported across studies and could hinder the reproducibility and external validation of candidate biomarkers.

Despite the promising results, the clinical applicability of the identified biomarkers remains limited by the need for external validation. Studies conducted by Wang et al. [7] and Dubey et al. [19] highlighted the importance of validating metabolomic signatures across diverse populations and clinical settings. Therefore, multicenter and longitudinal studies are required to establish the reproducibility, temporal stability, and clinical utility of these biomarkers. Additionally, the limited consideration of factors such as physical activity levels, which are known to influence metabolic pathways, represents an important knowledge gap that should be addressed in future investigations.

Although this study followed the methodology based on the Joanna Briggs Institute guidelines for scoping reviews, its nature imposes important limitations. As a scoping review, quantitative assessments, such as effect size analyses, were not performed. Additionally, this review did not assess the quality of the included studies or restrict the types of study designs, which should be considered when the conclusions about spondylitis are interpreted.

## Conclusions

Metabolomics shows promise for the identification of biomarkers in axSpA, enabling the distinction between patients and healthy controls while deepening the understanding of disease mechanisms. Amino acids, fatty acids, and metabolites involved in energy and lipid metabolism emerge as potential clinical discriminators. These metabolic signatures may contribute to the development of more effective therapeutic strategies. The modulation of metabolic profiles by TNF inhibitors underscores the importance of dysregulated metabolic pathways in disease progression and treatment response, supporting personalized management approaches. However, clinical implementation requires validation in large and diverse cohorts, standardization of analytical methods, and consideration of individual variability, including comorbidities. Overall, metabolomics represents a valuable tool for advancing biomarker development and precision medicine in axSpA.

## Data Availability

No datasets were generated or analysed during the current study.

## Abbreviations

AS: Ankylosing Spondylitis
ASDAS: Ankylosing Spondylitis Disease Activity Score
ASDAS-CRP: Ankylosing Spondylitis Disease Activity Score based on C-reactive Protein
axSpA: axial spondyloarthritis
BASDAI: Bath Ankylosing Spondylitis Disease Activity Index
ESR: Erythrocyte Sedimentation Rate
GABA: Gamma-aminobutyric acid
GC‒MS: Gas chromatography‒mass spectrometry
HC: Healthy controls
HLA-B27: Human leukocyte antigen B27
LC‒MS: Liquid Chromatography‒Mass Spectrometry
LDL: Low-density lipoprotein
LPCs: Lysophosphatidylcholines
MHC: Major histocompatibility complex
MS: Mass spectrometry
NAG: N-acetyl glycoproteins
NMR: Nuclear magnetic resonance
NSAIDs: Nonsteroidal Anti-Inflammatory Drugs
OA: Osteoarthritis
PCC framework: Population, Concept and Context
Phe/Tyr: Phenylalanine/tyrosine
PCs: Phosphatidylcholines
PRISMA-ScR: Preferred Reporting Items for Systematic reviews and Meta-Analyses extension for Scoping Reviews
RA: Rheumatoid arthritis
ReA: Reactive Arthritis
SCFA: Short-chain fatty acid
TNF: Tumor Necrosis Factor
uSpA: undifferentiated Spondyloarthritis
VLDL: Very-low-density lipoprotein
